# Applying an F-words lens to planning intervention for a child with autism: a retrospective case report with prospective follow-up

**DOI:** 10.3389/fpsyt.2026.1905890

**Published:** 2026-08-31

**Authors:** Pragashnie Govender, Roslyn W. Livingstone, Álvaro Hidalgo-Robles, Riclef Schomerus, Hércules Ribeiro Leite, Ginny Paleg

**Affiliations:** 1 Discipline of Occupational Therapy, School of Health Sciences, University of KwaZulu-Natal, Durban, South Africa; 2 Occupational Science and Occupational Therapy, Faculty of Medicine, University of British Columbia, Vancouver, BC, Canada; 3 Facultad de Educacion, Universidad Internacional de La Rioja, Logroño, Spain; 4 Research Unit ‘Participation with Impaired Physical and Motor Development’, Technische Universität (TU) Dortmund University, Dortmund, Germany; 5 Graduate Program of Rehabilitation Sciences, Universidade Federal de Minas Gerais, Belo Horizonte, Brazil; 6 CanChild Centre for Childhood Disability Research, McMaster University, Hamilton, ON, Canada

**Keywords:** autism spectrum disorder, developmental central hypotonia, hypermobility, F-words, F-words lens tool, hyperlexia, riding, masking

## Abstract

Children with Autism Spectrum Disorder (ASD) present with complex, multi-domain functional challenges that single-domain intervention approaches fail to address adequately. This retrospective case report, with prospective follow-up from age 7 years, applies CanChild’s F-words and the F-words Lens Tool to examine intervention planning and developmental outcomes across a five-year trajectory for an eight-year-old autistic girl with co-occurring hypotonia and joint hypermobility. For each developmental period, from 24 months to eight years, we identify the functional challenge encountered, drawing on standardized assessment data (CELF Preschool, Renfrew Action Picture Test, ADOS-2) alongside clinical and family report, and describe how F-words thinking informed clinical decision-making in real time; the F-words Lens Tool, developed subsequently, is applied here, retrospectively for earlier periods and prospectively from age 7, to structure this reasoning transparently, specify the interventions selected and their cross-domain rationale, and report outcomes. The case traces the child’s trajectory through the early developmental period, and following transition to mainstream schooling with presenting strengths and challenges. Caregiver accounts (father, grandmother) and the child’s own first-person perspective provide the lived experience outcomes across F-words domains. Together, this triangulated account demonstrates how the F-words and F-words Lens Tool can enhance clinical decision-making and family collaboration while preserving child agency, contributing an example to the global discourse on evidence-based, family-centred practice in complex neurodevelopmental presentations.

## 1 Introduction

Autism Spectrum Disorder (ASD) is a heterogeneous neurodevelopmental condition characterised by variability across multiple domains of functioning (1–4). This heterogeneity extends beyond differences in social-communication profiles to include motor function (5–7), sensory processing (8), and the functional consequences of co-occurring conditions such as developmental central hypotonia (9), associated with non-degenerative central causes, and joint hypermobility (10). These domains interact across development in ways that shape participation in everyday life (11–13), and cannot be adequately addressed by intervention approaches that target one domain in isolation.

The challenge in ASD clinical practice is not only *what* to address, but *how to think* about the child as a whole. When clinicians organise their reasoning around deficits and diagnoses, asking *what is wrong and how do we fix it?* they risk producing technically correct outcomes that fail to translate into meaningful participation; which can lead to isolated skills improving but everyday functioning, relationships, and overall wellbeing remaining unchanged.

The World Health Organization (WHO) International Classification of Functioning, Disability and Health (ICF) offers an alternative organising framework that encompasses body functions and structures, activities, participation, and contextual factors and positions health as the capacity to participate meaningfully across life domains, rather than the absence of impairment (14). Applied to childhood disability, the ICF encourages clinicians, families, and researchers to hold the child; their strengths, relationships, environment, and aspirations, in view simultaneously. Historically, however, the ICF has been difficult to apply accessibly in clinical practice or to communicate with families (15).

The CanChild F-words for Child Development (15, 16) address this challenge directly, offering a deliberately accessible, family-friendly language that brings the ICF’s way of thinking about health to life in clinical conversations, goal-setting, and everyday care (15, 16). Each F-word corresponds to a dimension of the ICF: Functioning (activities and participation), Family (the essential environmental context), Fitness (body functions, encompassing physical *and* mental wellbeing), Fun (engagement and enjoyment as participation), Friend(ship) (social participation), and Future (aspirations and life-course orientation) (15, 16). Together they shift the clinical question from *what is wrong?* to *who is this child, what do they need, and what do they and their family care about?*

The F-words Lens Tool (17, 18) extends this by providing a structured clinical reasoning instrument. By integrating the Rehabilitation Treatment Specification System (RTSS) (19) with the F-words (15, 16), it enables clinicians and families to map the specific *ingredients* of each intervention (what the clinician/family provides or manipulates), their hypothesised *mechanisms of action* (how they work), and the *targets* and *aims* they serve; with aims expressed as F-words participation goals. This transforms F-words (15, 16) thinking from an orientation into a clinical formulation to be communicated in plain language to families, scrutinised, and co-owned. Despite growing application of the F-words (15, 16) in cerebral palsy and other childhood disability contexts (20, 21), their application to ASD has received limited attention.

This case report applies the F-words (15, 16); as an ICF-based way of thinking about a child’s life; and the F-words Lens Tool (17, 18) to examine intervention planning and developmental outcomes across a trajectory for an autistic child with co-occurring hypotonia and joint hypermobility, followed from age 24 months to eight years. For each developmental period, the analysis identifies the primary functional challenge, describes how F-words thinking (15, 16) informed the family and clinical team’s planning in real time throughout this period; the F-Words Lens Tool (17, 18), developed subsequently, is applied here retrospectively to structure and make transparent the reasoning behind the interventions selected at each developmental stage, and prospectively from age 7 years, and to report outcomes.

## 2 Materials and methods

A systematic analysis of clinical records, standardised assessments, and family documentation covering the child’s development between ages three and eight years was undertaken. The case was reconstructed retrospectively from clinical and family documentation spanning the period from 24 months to 8.5 years of age. Information was derived from formal assessment and intervention records from treating therapists and other clinicians, including a paediatric neurologist and play therapist, as well as progress notes, reports provided by the family to treating professionals, and observations and experiences documented by the child’s mother during the course of the child’s development. The available clinical and family documentation was subsequently reviewed by the authors for the purposes of this case report. A family self-reported perspective was obtained when the child was 8 years old, and the child’s own account of her lived experience was obtained at 8.5 years by the authors. From approximately 7 years of age, the F-Words Lens Tool (17, 18) was applied prospectively to support structured clinical reasoning and intervention planning by specifying F-words goals, intervention ingredients, hypothesized mechanisms of action, targets, and examples of interventions. Ethical principles of informed consent and verbal assent from the child were upheld. Figures were generated using OpenAI’s ChatGPT image-generation tools, based on author-provided clinical data, interpretations, and participant-derived descriptions, and were subsequently reviewed for accuracy and presentation.

### 2.1 Description of the child and context

The child is an 8.5-year-old autistic female living with her extended family in a middle-income country in Africa, within a middle-class household. She was born at 35 weeks gestation following a pregnancy complicated by gestational diabetes, with birth weight of 3.03 kg and APGAR scores of 9/10 and 10/10. Early developmental milestones, walking (10.5 months), first words (10 months), and toilet training (completed before 24 months) fell within normative ranges. Developmental regression was first identified by the family, and prompted them to seek clinical evaluation. Expressive vocabulary plateaued at approximately 50 words around 24 months, alongside gradually intensifying echolalia, emerging sensory processing changes, refusal to sit on a toilet, resulting in a return to diapers and reduced social engagement, which became more apparent over the following weeks and months. No genetic testing has been pursued to date; known genetic aetiologies for the child’s presentation are therefore undetermined. At age 6 years 2 months, the child presented with trauma responses related to her father’s injury and the death of a close uncle, addressed through psychology/play therapy focused on emotional processing and safety.

### 2.2 Standard assessment profile and F-words mapping

**Figure 1:**
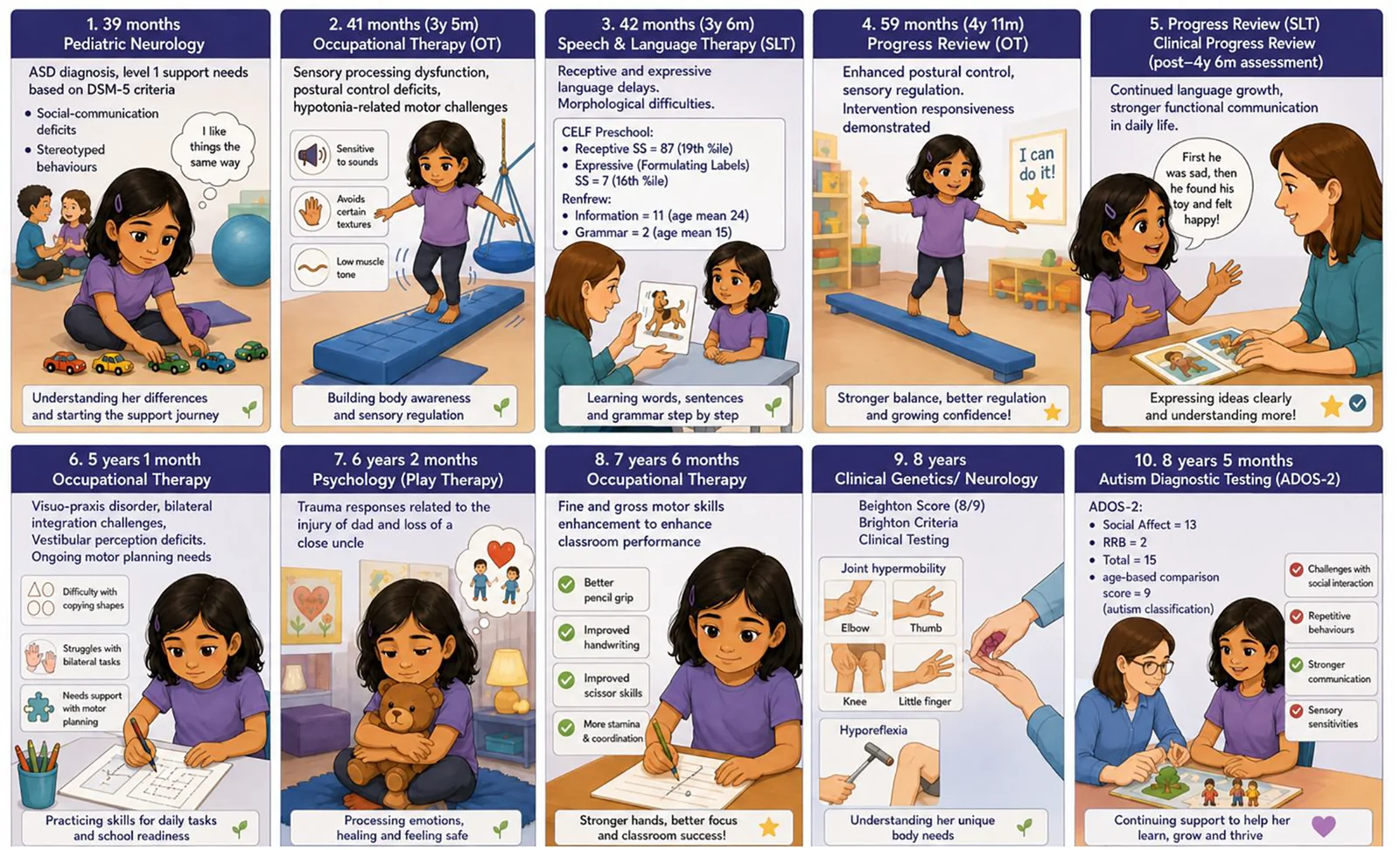
Assessment timeline.

Serial assessments from 39 months to 8 years 5 months established the child’s neurodevelopmental profile across domains relevant to the F-Words (15, 16) (see **Figure 1** for the assessment timeline).

Key findings were:

#### 2.2.1 Functioning

Occupational therapy evaluations identified sensory processing dysfunction (Sensory Processing Measure-P (22); DeGangi-Berk (23); Evaluation in Ayres Sensory Integration (24–26)), postural control deficits, hypotonia-related motor impairments, and later visuo-praxis and bilateral integration difficulties. Speech and language assessments revealed initial receptive and expressive language delays with morphological weaknesses. The CELF Preschool (27) at 42 months indicated below-average receptive language (std score = 87, 19th percentile) and expressive language (formulating labels std score = 7, 16th percentile). Renfrew Action Picture Test (28) scores corroborated these findings (information score = 11, age mean = 24, range 19–27; grammar score = 2, age mean = 15, range 8–19). Following weekly speech-language therapy sessions of 45 minutes over one year, reassessment at age 54 months (and reported at 59 months) demonstrated measurable improvement. CELF Receptive Language std score = 96 (39th percentile); CELF Expressive Language (formulating labels std score = 9; 37th percentile; recalling sentences std score = 7; 16th percentile, remaining below average). Renfrew scores similarly improved (information score = 24, age mean = 29, range 25–32; grammar score = 16, age mean = 21, range 17–25).

#### 2.2.2 Family

The diagnostic process was experienced by the family as poorly supported, with limited professional guidance following diagnosis. The family subsequently developed adaptive home strategies, structured routines, and multi-generational caregiving approaches independently, as detailed in Section 3.4.

#### 2.2.3 Fitness

Clinical evaluation using the Beighton Score (29, 30) and Brighton Criteria (30, 31) identified joint hypermobility syndrome. Hypotonia was identified as a contributing factor to postural and motor deficits across assessments. Early indicators of anxiety and emotional dysregulation, including somatic signs (palpitations, excessive sweating), rapid shifts between affective states, and escape/avoidance behaviours (withdrawing, seeking to hide), also informed fitness considerations and reflected mental health as well as physical wellbeing as central to this F-word.

#### 2.2.4 Friendships and fun

Assessment of social interaction and developmental screening confirmed ASD Level 1 support needs (DSM-5-TR) (1) with ADOS-2 (32) findings at 8.5 years (classification (Social Affect score = 13; Restricted and Repetitive Behaviours score = 2; total score = 15; age-based comparison score = 9, aligned to the cut-off for autism classification) (2.4) indicating persistent social-communication differences and restricted/repetitive behaviours, coexisting with emerging peer relationships and strong child-identified interests in physical and creative activities.

#### 2.2.5 Future

ADOS-2 (32) confirmed autism classification with high symptom severity, indicating ongoing support planning, situating this child’s development within a life-course perspective (33).

## 3 Applying the F-words: functioning challenges and intervention responses

The F-words (15, 16) provided the organising logic for analysing functional challenges, clinical reasoning, interventions and outcomes. Where the child or family expressed preferences or goals, these are foregrounded as evidence of the framework in action, not background context.

### 3.1 Early childhood (24–60 months): establishing functioning, fun, and family foundations

#### 3.1.1 Functioning challenge

By 26 months, the child presented with speech regression, including echolalia and jargon, sensory-seeking behaviours (crashing, jumping, visual fixation), and progressive social withdrawal, prompting the family to consult a speech-language therapist. No formal assessment was conducted and the family was advised that the presentation might reflect reduced language exposure during COVID-19 lockdown restrictions rather than a primary speech-language concern, and was counselled to continue engaging the child in language-rich activities. Given the regression and delays later confirmed by standardized assessment, this advice likely delayed formal evaluation and earlier intervention. Occupational therapy assessment at 41 months confirmed sensory processing difficulties and postural control deficits; and speech-language assessment at 42 months identified expressive and pragmatic language delays, through standardized testing (reported above). The central functioning concern was not isolated skill deficit, but disruption to daily participation: verbal communication, alongside self-care and play, was affected (core occupations of early childhood). However, several developmental strengths were present at this stage: early hyperlexic traits (spontaneous letter and word recognition) (34–36), a strong drive toward physical and creative play, and clear preferences for specific sensory experiences, including deep pressure and rhythmic movement. These assets, were reflected in the F-words-informed thinking that guided planning at this stage.

#### 3.1.2 F-words reasoning

Applying F-Words thinking at this stage meant asking: what does this child need to function in everyday life, and what does her family need to support that?

The family’s goal: “to raise a happy and healthy child” anchored planning within the family’s values rather than the clinician’s agenda. Fun was explicitly incorporated as a non-negotiable criterion: activities had to be child-directed and intrinsically motivating, because sustained engagement, not short-term compliance, was considered to produce generalized developmental gain. The child’s self-initiated sensory-seeking behaviours were recognised as early expressions of functioning and directed toward the interventions most likely to be effective (37–39). Fitness, encompassing both physical wellbeing and emerging emotional regulation needs, shaped the selection of low-impact, sensorimotor-rich activities. A future orientation was explicit rather than assumed: the choice of early interventions (40) reflected a deliberate investment in foundations for lifelong participation.

#### 3.1.3 Interventions and rationale

Three core interventions were initiated, and aligned with a cross-domain reach across the F-words. F-words thinking also informed real-time evaluation of the evidence base for each intervention as it was being considered, helping ensure resource allocation reflected available research. This included evidence for equine-assisted therapy, dance and music, and aquatic intervention, which provided grounding for multi-modal decisions the family could evaluate within their own context and values.

Occupational Therapy using Ayres Sensory Integration^®^ (ASI^®^) was delivered through weekly child-directed therapy sessions, supplemented by a home sensory diet and school accommodations. Graded sensory challenges within child-initiated, meaningful activity were presented to facilitate sensory processing through adaptive responses (37, 39) and improved sensory modulation and motor planning during functional tasks. The F-Words aims spanned functioning (sensory regulation, postural control), fun (child-directed engagement, intrinsic motivation), and family (home sensory diet embedded in caregiving routines). The child’s own sensory-seeking behaviour was used as the starting point for activity selection and application of child agency within the fun domain. DIRFloortime^®^ was integrated to foster shared attention and emotional reciprocity, centering caregiver-child interaction as the mechanism of change (41) an approach explicitly aligned with the F-word family.

Speech-language therapy addressed communicative functioning through echolalia reduction and pragmatic language development, with strategies embedded in natural family routines (family) and family-facilitated peer-dyad sessions (early friendships).

Swimming, introduced within this period for its low-impact strengthening properties given co-occurring hypotonia, was also identified as a child-preferred activity based on observed enjoyment with water play, and it simultaneously addressed fitness, functioning (vestibular-proprioceptive input, postural control), and fun (42, 43).

#### 3.1.4 Outcome

By 59 months, improved sensory regulation and bilateral integration were observed across functional settings. Speech-language abilities reached age-average performance, reflecting both intervention response and developmental maturation. Critically, these gains were not confined to clinical sessions: the grandmother reported observable improvements in concentration and affect during activities. This convergence between therapy goals and lived family experience illustrates that F-words was aligned with what the family actually valued.

### 3.2 School age (60–96 months): navigating participation, friendships and masking

#### 3.2.1 Functioning challenge

Entry into mainstream schooling followed a period of good progress; speech-language therapy and occupational therapy services concluded at this transition on the basis that the child was assessed as functionally ready for grade one. Ongoing monitoring was provided through the educators and school developmental support team (therapists). The child’s educators were not specifically trained in ASD but had received general training in supporting neurodiverse students, consistent with the privately-funded school context. Alongside a strengthening academic profile (hyperlexia now leveraged for literary achievement), two clinically significant patterns emerged. First, the child engaged in sustained masking; the conscious suppression of ASD-related traits to appear socially conforming (44). Masking and its associated threats to wellbeing (social anxiety, burnout, cognitive load) were identified through parental observation, and by behavioural changes at home, (meltdowns, withdrawal, some verbal expressions indicating distress); however no standardized measure was used given the child’s young age and limited capacity to articulate her internal experience at this stage.

#### 3.2.2 F-words reasoning

The masking pattern presented an F-words tension (short-term friendships gained through social conformity versus long-term future wellbeing) F-Words reasoning required the team to hold these competing domains overtly rather than resolving the tension by prioritizing one at the expense of the other. Through reflecting on the clinical question from *how do we reduce disruptive behaviour?* to *how do we support this child’s capacity to participate and regulate across contexts without compromising her developing identity and mental health*?, the issues were reframed.

This reframing required recognizing the child’s self-selected activities, identified through direct observation of what she consistently gravitated toward, spontaneous verbal expression of interest, and unprompted mimicry, not as preferred leisure but as F-words assets with multi-domain reach. Swimming, riding, and dance simultaneously addressed fitness (physical conditioning, strength), functioning (sensory regulation, motor planning), fun (intrinsic enjoyment and self-expression), and friendships (structured peer interaction within shared activities). This was a key demonstration of the F-Words in action: rather than asking *which of these activities is the most therapeutic?* F-Words reasoning asked *which activities serve which F-Words domains simultaneously, and how can we protect them?*

The family F-word was actively present in the father’s evolving approach, namely, his self-described transition from managing behaviour to creating calm, responsive environments and prioritizing listening reflects a shift from corrective to responsive parenting. Rosenbaum and colleagues (33) position family as the essential context and content of childhood development; the father’s account demonstrates this is not merely a theoretical claim but a description of how therapeutic change actually moves through family relationships. His eventual recognition that he may share some neurodevelopmental traits/differences himself adds a further dimension to family-centred reasoning: the family system is not a fixed, neutral backdrop but a dynamic network of strengths, needs, and learning.

#### 3.2.3 Interventions and rationale

##### 3.2.3.1 Riding

This was continued from early childhood into the school-age period. Through engagement, combined vestibular-proprioceptive integration (supporting sensory functioning), postural demand/core stability (fitness), and the relational demands of horse-rider communication as a scaffold for verbal interaction were considered constituting an early bridge to social engagement) (45, 46).

##### 3.2.3.2 Dance

Independently initiated by the child at 4.5 years, observed through spontaneous dancing in front of mirrors prior to any formal instruction, this presented a clinical dilemma: *how to reconcile joint hypermobility with participation in dance*? The F-words reasoning process that resolved this dilemma is worth making explicit. A deficit-focused assessment would ask: *does this activity carry unacceptable physical risk? Whereas the* F-Words reasoning asked *what does this child stand to gain across multiple F-Words domains if participation can be made safe, and does that cumulative gain outweigh the managed risk?*

Through dance, F-words aims spanned fun (intrinsic enjoyment and child-initiated engagement, a key predictor of sustained participation), functioning (bilateral coordination, body awareness), friendships (scaffolded peer interaction in a structured social context), fitness (strength, flexibility within safe parameters), and future (creative self-expression as a regulatory and identity-building resource into adolescence) (47, 48). Risk mitigation via appropriate footwear, instructor education, activity modification (49); enabled rather than restricted participation. The child’s personal preference was treated as F-words evidence, not as a preference to be weighed against clinical judgment.

##### 3.2.3.3 Hyperlexia as F-words asset

Hyperlexia (34–36) was systematically developed as an F-words resource. Academically, it supported functioning in literacy and knowledge acquisition. Communicatively, it provided an alternative pathway for expression when verbal communication was effortful. Creatively, it was developed into writing as an outlet for emotional processing (fitness) and fun, and eventually as a vehicle for friendships (50, 51).

#### 3.2.4 Outcome

Progression across motor milestones was sustained despite hypotonia and joint hypermobility: based on descriptive and functional observation rather than standardized motor assessment, swimming advanced from water safety to competitive participation; riding from assisted balance to independent trotting; dance from structured motor learning to creative expression. Social-communication development progressed from parallel play to emerging reciprocal peer relationships, as reported by parents and the classroom educators. No standardized measure of peer interaction was administered. Engagement across all interventions was consistently good throughout, with no reported adherence difficulties. No adverse events, complications, or unanticipated events were recorded in association with any intervention over the course of care. The family system evolved from crisis response to informed advocacy; a family outcome of equal developmental significance to the child’s individual gains.

### 3.3 Current status (96–101 months): sustaining participation and future planning

#### 3.3.1 Functioning challenge

At just over eight years, the child sustains academic participation in a mainstream curriculum with strengths in literacy and creative arts. Persistent challenges include performance anxiety, episodic burnout attributable to masking demands, emotional dysregulation, and emerging somatic pain complaints, the latter warranting evaluation in the context of ASD-associated interoceptive differences and evolving joint hypermobility syndrome (fitness). Peer relationships (friendships) remain challenging, particularly in domains of social perception and perspective-taking. The central functional question at this stage concerns future: *how do current intervention choices build foundations for sustainable participation, wellbeing, and identity through adolescence, not merely within the current developmental period?* This forward orientation is not a distal aspiration, but active planning shaping current decisions; a perspective the child herself articulates directly: *‘I’m different, but I can still make a difference in this world, and so can you’* reflecting an emerging self-concept that integrates difference with agency and future-oriented purpose.

#### 3.3.2 F-words reasoning and formal lens tool application

**Figure 2:**
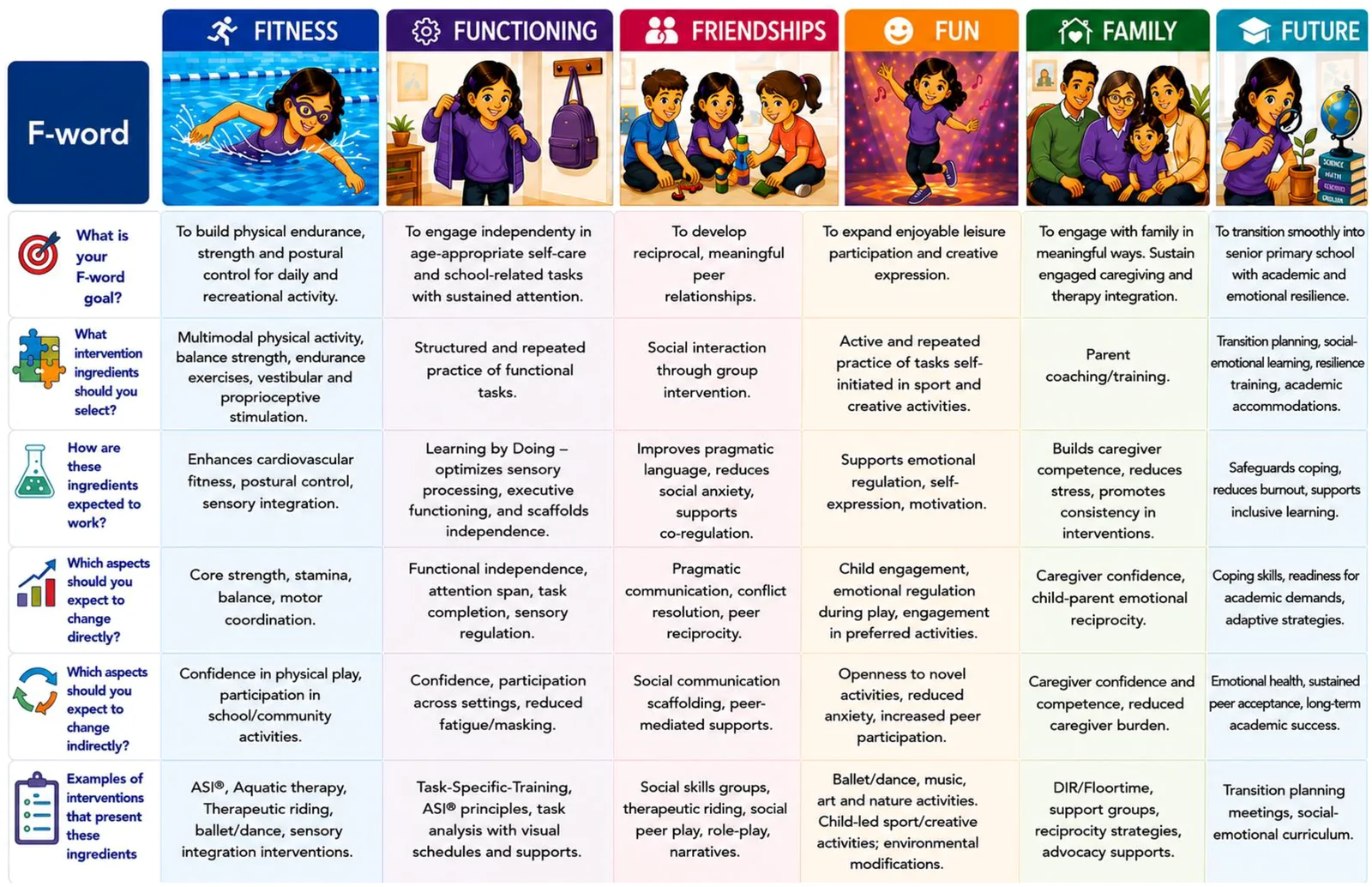
The F-words lens tool: identifying intervention components (adapted from Leite et al., 2024).

From 7.5 years, the F-words Lens Tool (17, 18) was applied prospectively, combining the RTSS (19) with the F-Words (15, 16) to map intervention ingredients across domains (see **Figure 2**). The F-Words Lens Tool (17, 18) formalised what had previously been implicit across Sections 3.1 and 3.2, namely, the multi-domain F-words reach of each intervention, expressed in RTSS (19) language.

For each intervention, the *ingredient* is specified (what the clinician provides), its hypothesised *mechanism of action* (how it works), the *target* (the measurable change expected directly), and the *aim* (the broader F-words outcome). Unlike informal F-words labelling, the Lens Tool (17, 18) requires clinicians to specify how each intervention ingredient is expected to produce a particular F-words outcome, making the reasoning auditable and open to family scrutiny.

This process was carried out *with* the family in plain language. Presenting the F-words Lens Tool (17, 18, 52) analysis in accessible language enabled the family to evaluate the rationale behind each intervention; including activities that appeared to carry physical risk, and to prioritise based on their own values and observations. The family became co-reasoners in the process. This reflects the F-words core positioning of family as active partners in health decision-making (15, 16, 33).

A future orientation now actively shapes transition planning. Particular attention is directed toward masking in adolescence (53–56). This can be done through the protective assets already established: physical fitness routines providing regulatory and relational stability, creative outlets (writing, dance) supporting identity and emotional expression, and a family system now equipped with the understanding and advocacy skills to navigate the increasing demands of adolescence.

### 3.4 Family and child perspectives

#### 3.4.1 Family perspectives

**Figure 3:**
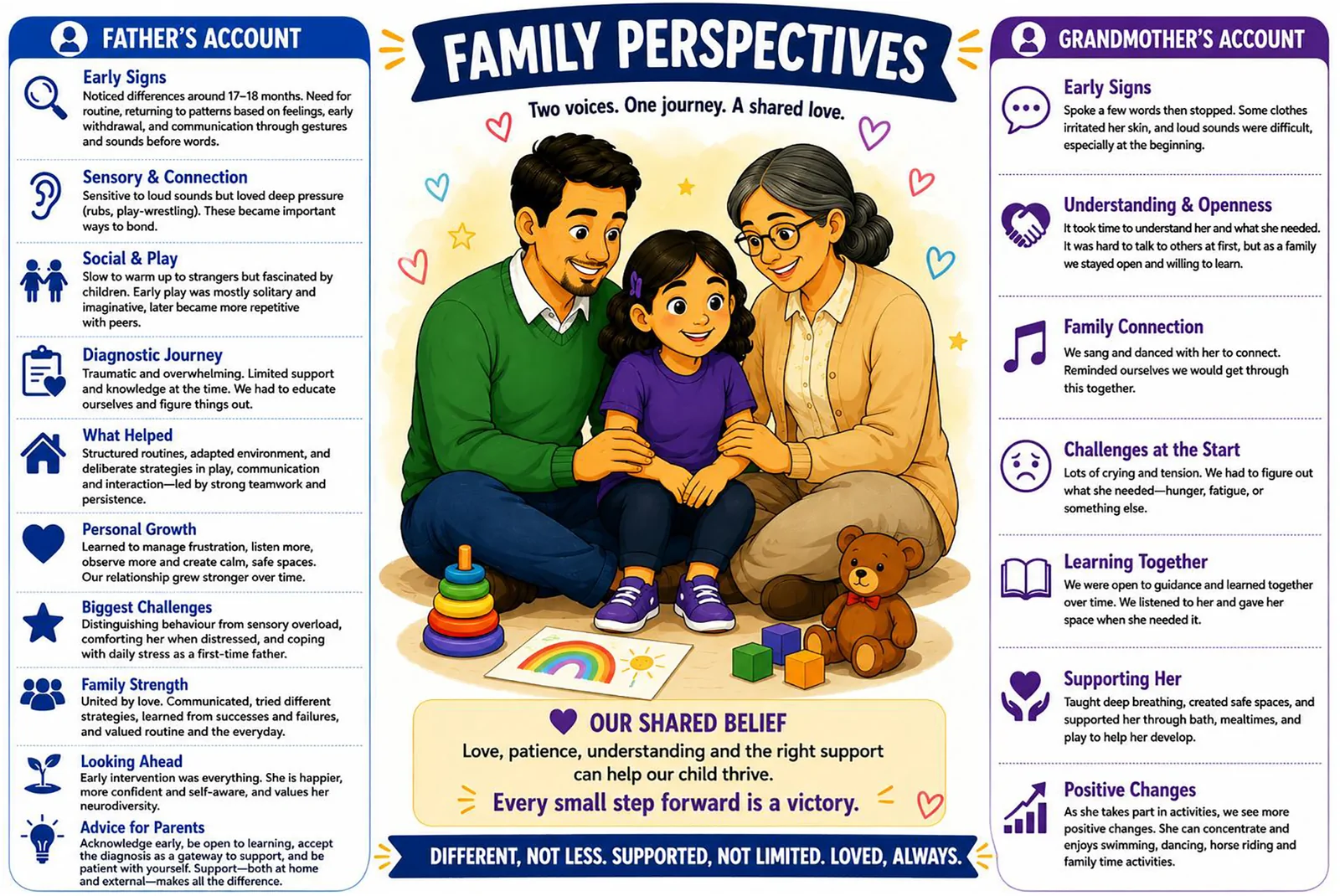
Summary of caregiver accounts.

The caregiver accounts (summarized in **Figure 3**; full accounts in **Appendix 1**) provide an important form of ecological validation. The grandmother, without recourse to clinical frameworks, arrived at observations that map directly onto F-words domains. She noted that participation in swimming, dancing, and horse riding produced visible improvements in concentration and affect simultaneously, providing a lay articulation of multi-domain F-Words impact. The father’s account traces a trajectory from post-diagnostic disorientation and inadequate professional support to informed advocacy and adaptive family practice, a family outcome central to the framework’s goals. His description of his daughter as *“a happier, a more confident, and more self-aware child and she has learnt to value her neurodiversity”* maps onto the F-words constructs of fun, functioning, and future in the language of lived experience rather than clinical measurement. These accounts support the F-Words ecological validity and its alignment with values families hold when adequately supported.

#### 3.4.2 Child’s perspective

The child described both the challenges she experiences and the activities that help her navigate them. She explained that *“sometimes I feel very tired … I feel a little jelly,”* acknowledging that everyday things *“are hard for me, like waking up on time and putting where everything goes and trying everything to be neat in my head.”* Despite these challenges, she repeatedly returned to the activities she loves and the joy they bring her. Speaking about dancing, she shared, *“I have a fun passion for it … even when I’m tired, I never ever stop.”* Swimming was described as a place where she feels better emotionally: *“Every time I feel down, I just be happy when I swim because it’s calming for me and … it helps me focus.”* She also described horse riding as something that *“helped me keep my posture,”* adding that she loved *“the jumps that we did.”*

As she reflected on herself, she acknowledged, *“I’m different,”* before expressing confidence and hope: *“I’m different, but I can still make a difference in this world, and so can you.”* She described her strengths in reading, music, painting and drawing, and spoke proudly about becoming captain of her swimming team, earning *“lots of ribbons and medals,”* and achieving good grades. She also viewed learning itself as an achievement: *“Sometimes it can be very little things like learning a new song on the piano and a new dance move,”* adding that, *“more ideas just flow in my head, sometimes too many and I can’t stop.”*

Taken together, her narrative portrays a young girl who openly acknowledges that some things are difficult, yet consistently defines herself through her passions, strengths, curiosity, and determination. Her own words reveal that meaningful activities are not simply hobbies but ways of feeling calm, expressing herself, building confidence, and imagining a future in which, despite being *“different,”* she believes she *“can still make a difference in this world.”*

### 3.5 Protective factors and risks: a lifespan F-words perspective

A life-course perspective is essential to childhood-onset disability, enabling consideration of how support translates across developmental stages (33). The F-words (15, 16) support this perspective by continuously balancing current participation with future wellbeing. In this case, protective factors included early identification leveraging neuroplasticity (40), established physical activity t, cognitive strengths (34–36), and a supportive family system. Key risks requiring ongoing monitoring include the cumulative psychological cost of masking (56); performance anxiety and increasing social demands as peer relationships develop (56). The F-words lens positions future as an active planning domain rather than a distal aspiration, supporting continued attention to these risks while the child’s regulatory and relational foundations remain strong.

## 4 Discussion

### 4.1 The F-words as a clinical reasoning tool

The analysis demonstrates that the most effective interventions in this case consistently addressed multiple F-words domains simultaneously. The shift from *‘what deficit needs fixing?’* to *‘across which F-Words domains can this intervention contribute?’* produced a pattern of intervention synergy which would not have been adequate achieved via a single-domain assessment. The F-words Lens Tool (17, 18) assisted in this multi-domain analysis. Swimming illustrates this multi-domain reach, addressing fitness, functioning and fun while supporting friendships and future participation. Rendering this analysis visible to the family, in plain language, enabled them in resource allocation decisions and to observe and report on developmental changes that aligned with targets. Viewing the family as the critical environment in which development occurs, and as a focus of intervention in its own right, reflects emerging thinking in childhood-onset disability (33).

### 4.2 Child agency and the F-words

Child-initiated activities can support sustained engagement and generalization beyond the therapy setting. In this case, dance, initiated and sustained by the child, provided benefits across multiple F-words domains and illustrates the role of intrinsic motivation in participation and skill development (57, 58). The F-words formalized what the family had already recognised: fun was not simply a leisure consideration but an important contributor to sustained engagement and participation. It also provided a structured rationale for supporting child-preferred activities while managing associated risks. Child agency was also reflected in the family context. The father described a shift from managing and redirecting behaviour toward listening, observation, and creating environments in which his daughter could express herself. This reorientation toward responsive parenting further positioned the child as an active participant in her development.

### 4.3 Evidence-based practice and family F-word

The family F-word was operationalized at three levels: embedding intervention strategies within everyday family routines (bathtime, mealtimes, play); recognizing caregiver knowledge and attunement as clinical material; and building the family’s capacity to advocate and adapt independently of professional support (59).

This transparency in reasoning, later formalized through the F-words Lens Tool, was possible because family-centred reasoning was already embedded in real-time decision-making. The family evolved from recipients of professional advice to active co-designers of intervention, with caregiver observations contributing to intervention selection and adaptation. The child’s account further supported these caregiver perspectives across multiple F-words domains: her descriptions of activities as sources of calm, focus, posture, and pride reflect fitness and fun; her pride in academic achievement reflects functioning; her account of team captaincy and earned recognition; and her explicit acknowledgment of difficulty alongside a clearly articulated, future-oriented sense of self reflects the same holistic balance across domains that the F-words framework aims to capture.

### 4.4 Co-occurring conditions and the F-words

Children presenting with ASD and co-occurring conditions require careful balancing of therapeutic benefit against risk. The F-words supported this balance by providing a structured multi-domain rationale: when an activity offered substantial fun, functioning, fitness, and friendships benefits simultaneously, the threshold for accepting managed risk was supported by a cumulative F-words argument that would not have been articulable through single-domain analysis alone. Dance participation is the clearest example: a conventional deficit-focused assessment might have concluded *‘contraindicated due to hypermobility’*; F-words reasoning asked *‘can we make this safe enough to capture the multi-domain gain?’*. Masking-related burnout illustrates why fitness must be read as inclusive of mental wellbeing (15): naming burnout within this domain gives it equivalent clinical weight to the physical risks of dance or swimming, changing what is monitored and how it is acted on.

### 4.5 Long-term trajectory and the future F-word

The future F-word is structurally different from the other five: it is not a domain of current functioning but a temporal orientation and commitment to asking, at every decision point, *how current choices connect to longer-term participation, wellbeing, and identity* (15, 33). In practice, this means treating the regulatory strategies, physical activities, and creative outlets established in early childhood not as temporary therapeutic measures but as foundations to be protected and developed through subsequent transitions. This study used the F-words Lens Tool (17, 18) both retrospectively and prospectively, recognizing that it is a flexible and useful approach. It helps to understand whether previously planned interventions are using appropriate ingredients, as well as to prospectively involve families in the process of selecting and understanding the key ingredients and other important components of clinical reasoning. This will remain particularly important as the child approaches adolescence, when masking, mental wellbeing, and changing social demands require ongoing attention. above.

## 5 Limitations

This single-case retrospective analysis carries several inherent limitations. Findings reflect a specific child, family, and resource context; individual variability in ASD presentation, family resources, cultural context, and co-occurring conditions substantially shapes intervention outcomes, and these findings should not be generalized without care. The application of the F-words framework to early interventions (ages 3-7) relied on clinical record reviews and family recall, introducing potential bias. Prospective application from 7.5 years provided more systematic data but covers a shorter developmental window. The multi-modal intervention approach makes it impossible to attribute outcomes to specific interventions versus developmental maturation or general enrichment effects. Resource access is a significant limitation: this family’s ability to engage in riding, private swimming instruction, dance, and access multiple health services simultaneously is not representative of most families affected by ASD. Questions of equity and replicability in less-resourced contexts require explicit consideration. Cultural and linguistic factors were not systematically examined, limiting understanding of how family background and community context shaped intervention selection and implementation. The child’s perspective was gathered through an open conversation supplemented with self-initiated visual support (emotion and strengths imagery), appropriate to her age and communication profile; this approach, while enabling meaningful self-report, differs from validated child-report instruments and should be interpreted as illustrative. Long-term follow-up into adolescence and adulthood remains necessary to assess the sustained impact of the intervention trajectory reported here.

## 6 Conclusion

This case report demonstrates how the F-words and the F-words Lens Tool, can structure intervention planning for children with complex neurodevelopmental presentations. By making the central question *‘across which F-Words domains can we support this child’s participation?*’, the framework produced a coherent, multi-domain, and family-centred approach to five years of intervention planning. Three insights from this case have particular implications for practice. First, F-words reasoning enabled the acceptance; with appropriate risk mitigation; of activities that conventional deficit-focused practice might have restricted, capturing multi-domain developmental gains that justified the risk. Second, child-initiated interests, when incorporated within F-Words–guided parameters, produced sustained engagement and generalized gains across domains that extended well beyond the targeted skills Third, the F-words Lens Tool gave the family a shared language for understanding clinical decisions, enabling them to transition to more active, informed partners. That the family’s accounts map onto the framework’s constructs provides ecological validity for the F-words. Future research should examine the F-words framework’s (15, 16) application in ASD across diverse cultural and socioeconomic contexts, explore optimal implementation strategies for the F-words Lens Tool (17, 18) in family-clinician partnerships, and develop standardised outcome measures aligned with F-Words domains to strengthen the evidence base for participation-oriented intervention.

## Data Availability

The original contributions presented in the study are included in the article/supplementary material. Further inquiries can be directed to the corresponding authors.

## AppEndix 1 Detailed Caregiver Accounts

Father’s Account:

“I first noticed that something might be different in her development when she was around 17–18 months old. At the time, I did not fully understand her behaviours. Only later did it become clearer; particularly her need for routine and the way she would return to certain behaviour patterns depending on how she was feeling, whether sad, angry, or happy. One of the early challenges was her tendency to withdraw, both emotionally and physically. Before she developed stronger verbal skills, she communicated through body and hand gestures, along with sounds that seemed like she was talking, even though she was not using words. She also had significant sensory sensitivities. She could not handle loud noises, but at the same time, as a baby, she enjoyed body rubs or massages (deep pressure), which soothed her. I used to “play-wrestle” with her, and she really enjoyed it; it became an important way for us to bond. Socially, she did not take quickly to strangers, although she was fascinated by other children. Her early play was mostly solitary and imaginative, partly influenced by the COVID period, and later became more repetitive when interacting with peers. The diagnostic process was very traumatic for us as a family. We struggled to find professionals with sufficient knowledge of autism, and when we received the diagnosis, I felt terrified, angry, sad, and remorseful. At that time, I did not understand autism at all, and we were not given much support. We had to figure things out ourselves through research. Much of what we implemented as a family came from my wife’s efforts. She guided us in creating structured routines and adapting the home environment. Everything we did; how we played, communicated, and interacted; became deliberate, because we learned that this was necessary for her to understand and learn. It was very, very tough. One of my personal challenges was managing my own frustration, especially as a first-time father coming from a very different upbringing. Over time, I have learned a great deal. Initially, my understanding of autism was limited and shaped by misconceptions. I thought it was something that required constant medical care, but I was wrong. Through this journey, I have also reflected on myself and recognise that I may share some similar traits, although I was never diagnosed. One of the biggest challenges was trying to comfort her when she was distressed or overwhelmed. It was often difficult to distinguish between behaviour and sensory overload. In most cases, our immediate response was simply to try to calm her, even if we did not fully understand the cause. My approach to parenting had to change almost immediately after her diagnosis. Every day has been, and still is, a learning experience. I have learned to connect with her by creating calm, safe environments where we can be together, talk, and allow her to express herself. I have had to learn to listen more and observe more. Our relationship has improved significantly over time. It has been tough, but also very rewarding, and I continue to try my best as a father. As a family, we had no choice but to work together. Our love for her and our desire to give her the best drove us to learn quickly and adapt. We communicated regularly, tried different strategies, and learned through both successes and failures. Over time, we realized how important routine was; and appreciating that ‘the everyday’ mattered. Looking back, early intervention played a critical role in her development; if I am being honest, it was everything. Without it, I do not believe she would be where she is today. Now, she is a happier, a more confident, and more self-aware child and she has learnt to value her neurodiversity. While there are still challenging days, each day is a new learning experience for all of us. If I could share my learnings with other parents, it would be to acknowledge the challenges early, be open to learning, and accept the diagnosis as a gateway to assist and not limit their child. I would also encourage parents to be more patient with themselves and others during the process. This is not something that simply goes away; interventions both within the family and from external supports is what has helped our little girl.”

Grandmother’s Account

“In terms of early signs and concerns, she spoke in a few words and then stopped. This was scary and worrying for us. It took us time to really understand her and what she needed. We also noticed that some clothes irritated her skin, and loud sounds were difficult for her, especially in the beginning. It was hard at first to talk to people, even friends, about what we were experiencing. As a family, we tried to connect with her, we sang and danced with her, which helped at times. We all had to work together as a family and remind ourselves that we would get through this. At the beginning, there was a lot of crying and tension. We had to figure out what she needed; whether it was hunger, fatigue, or something else. We were open to learning and guidance on understanding how to support her needs, and we all learned together over time. We realized that we had to listen to her and also give her space when she needed it. At first, I didn’t know what to do. But as she is growing older, I am starting to understand her better and what should be done. I always tried to do things that would help her, such as teaching her deep breathing to keep her calm and supporting her through difficult moments and making her feel safe and providing opportunities during bathtime and eating and playtime to help her develop. As she began to take part in activities, I also noticed more positive changes. She is able to concentrate, and she seems happy doing things like swimming dancing horse riding and family time activities.”
